# Appropriate Activity Assays Are Crucial for the Specific Determination of Proline Dehydrogenase and Pyrroline-5-Carboxylate Reductase Activities

**DOI:** 10.3389/fpls.2020.602939

**Published:** 2020-12-23

**Authors:** Sandrine Lebreton, Cécile Cabassa-Hourton, Arnould Savouré, Dietmar Funck, Giuseppe Forlani

**Affiliations:** ^1^Sorbonne Université, UPEC, CNRS, IRD, INRAE, Institute of Ecology and Environmental Sciences—Paris, IEES, Paris, France; ^2^Department of Biology, University of Konstanz, Konstanz, Germany; ^3^Department of Life Sciences and Biotechnology, University of Ferrara, Ferrara, Italy

**Keywords:** proline dehydrogenase, pyrroline-5-carboxylate reductase, enzyme activity assay, electron acceptor, protein extraction, mitochondria

## Abstract

Accumulation of proline is a widespread plant response to a broad range of environmental stress conditions including salt and osmotic stress. Proline accumulation is achieved mainly by upregulation of proline biosynthesis in the cytosol and by inhibition of proline degradation in mitochondria. Changes in gene expression or activity levels of the two enzymes catalyzing the first reactions in these two pathways, namely pyrroline-5-carboxylate (P5C) synthetase and proline dehydrogenase (ProDH), are often used to assess the stress response of plants. The difficulty to isolate ProDH in active form has led several researchers to erroneously report proline-dependent NAD^+^ reduction at pH 10 as ProDH activity. We demonstrate that this activity is due to P5C reductase (P5CR), the second and last enzyme in proline biosynthesis, which works in the reverse direction at unphysiologically high pH. ProDH does not use NAD^+^ as electron acceptor but can be assayed with the artificial electron acceptor 2,6-dichlorophenolindophenol (DCPIP) after detergent-mediated solubilization or enrichment of mitochondria. Seemingly counter-intuitive results from previous publications can be explained in this way and our data highlight the importance of appropriate and specific assays for the detection of ProDH and P5CR activities in crude plant extracts.

## Introduction

**FIGURE 1:**
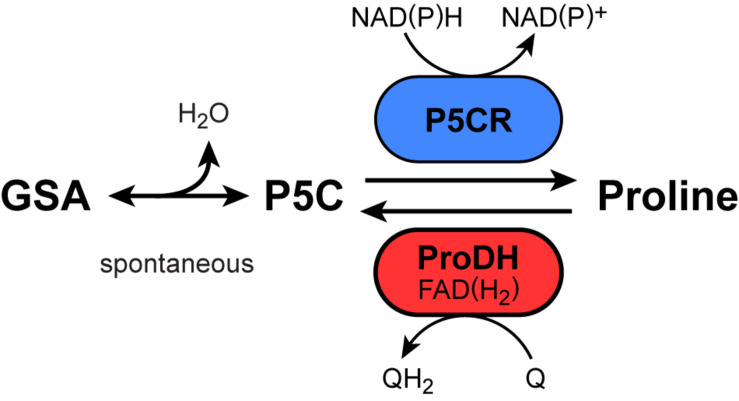
Reactions and enzymes known to produce or degrade proline in plants. GSA, glutamate-5-semialdehyde; P5C, pyrroline-5-carboxylate; P5CR: P5C reductase; ProDH, proline dehydrogenase, Q/QH_2_, oxidized/reduced ubiquinone.

Many prokaryotes and eukaryotes accumulate free proline as a compatible solute in response to abiotic or biotic stress (Forlani et al., 2019; Trovato et al., 2019). Therefore, in order to improve crop performance under stress conditions, a lot of research has been dedicated to understand the molecular basis of proline accumulation and the role of this amino acid in plant acclimation and stress tolerance (Szabados and Savoure, 2010; Forlani et al., 2019; El Moukhtari et al., 2020). Multi-level analyses and flux modeling demonstrated that different plant species rely on different regulatory mechanisms of either transcriptional regulation or modulation of enzyme activities to shift the balance between proline biosynthesis, consumption, and degradation toward proline accumulation (AbdElgawad et al., 2015; Hildebrandt, 2018; Dellero et al., 2020). Proline is synthesized from glutamate and also degraded to glutamate via the common intermediate glutamate-5-semialdhyde (GSA), which is in spontaneous equilibrium with pyrroline-5-carboxylate (P5C, Figure 1). Proline production is catalyzed by the sequential action of P5C synthetase and P5C reductase (P5CR), the latter being the only known enzyme to synthesize proline in plants (Trovato et al., 2019). P5CR can utilize both NADH and NADPH as the electron donor for P5C reduction (Giberti et al., 2014). Proline degradation occurs in mitochondria by the sequential action of proline dehydrogenase (ProDH) and P5C dehydrogenase (P5CDH; Trovato et al., 2019). Whereas P5CDH is a soluble enzyme in the mitochondrial matrix and uses NAD^+^ as the preferential electron acceptor (Forlani et al., 1997, 2015a), ProDH is a membrane bound flavoenzyme that seems to donate electrons directly to ubiquinone in the mitochondrial inner membrane (Elthon and Stewart, 1981; Servet et al., 2012; Schertl et al., 2014; Cabassa-Hourton et al., 2016). To account for the frequent observation of oxygen consumption in conjunction with proline oxidation, ProDH was and is occasionally also referred to as proline oxidase, but good evidence for direct electron transfer to molecular oxygen has not been obtained so far.

Most of the molecular data about proline metabolism has been obtained with Arabidopsis, but genes for proline metabolic enzymes have also been cloned from other model species and their expression has been analyzed. Numerous studies have analyzed the correlation between stress tolerance and proline content as well as transcript and activity levels of proline metabolic enzymes (Per et al., 2017). Two activities can be easily detected in crude extracts of soluble proteins from plants: P5C-dependent oxidation of NAD(P)H with a pH-optimum at 7–8 (with NADH) or 8–9 (with NADPH; Giberti et al., 2014), and proline-dependent NAD^+^-reduction at pH > 9 (Rena and Splittstoesser, 1975). Early purification attempts already indicated that both activities may be mediated by the same protein, i.e., P5CR (Rena and Splittstoesser, 1975), and later on purified P5CRs were shown to catalyze both reactions (Krueger et al., 1986; Szoke et al., 1992). Because P5C is unstable in neutral solutions and not commercially available, many subsequent papers used proline-dependent NAD^+^ reduction at high pH to assay P5CR activity, although it does not represent a physiological feature (Nocek et al., 2005; Meng et al., 2006).

Analysis of proline-dependent respiration in isolated corn mitochondria showed that the respiration rates were insensitive to NAD^+^ addition and that ProDH is bound to the inner mitochondrial membrane (Elthon and Stewart, 1981, 1982). Solubilization of membrane-bound proteins from corn or Arabidopsis mitochondria allowed the detection of ProDH activity as proline-dependent reduction of decylubiquinone, 2,6-dichlorophenolindophenol (DCPIP), cytochrome *c*, or iodonitrotetrazolium (Rayapati and Stewart, 1991; Schertl et al., 2014). These activities were nearly completely absent in mitochondria isolated from Arabidopsis *prodh1/prodh2* double mutants (Cabassa-Hourton et al., 2016). Structural and functional studies on ProDH from bacteria (PutA), yeast (Put1) or human showed that they all contain a tightly bound FAD cofactor and transfer electrons to a membrane-localized quinone (Wanduragala et al., 2010; Tallarita et al., 2012; Singh et al., 2014; Tanner, 2019).

Against this large body of evidence, following early reports claiming the occurrence of an NAD-linked “ProDH” in wheat (Mazelis and Creveling, 1974) and *Chlorella pyrenoidosa* (McNamer and Stewart, 1974), numerous publications have assigned proline-dependent NAD^+^ reduction at high pH to ProDH activity. Our detailed characterization of recombinant proteins, plant protein extracts, and isolated mitochondria from Arabidopsis wildtype and mutants clearly shows that only P5CR mediates this activity. ProDH activity can be specifically quantified as proline-dependent DCPIP reduction, but requires membrane-solubilizing agents for extraction or enrichment of mitochondria.

## Materials and Methods

### Plant Material and Cultivation Conditions

Arabidopsis [*Arabidopsis thaliana* (L.) Heynh.], ecotype Col-0 and T-DNA insertion lines were obtained from the NASC and the GABI-KAT project as described previously (Funck et al., 2010; Kleinboelting et al., 2012; Cabassa-Hourton et al., 2016). A *prodh1-4/prodh2-2* double mutant was obtained by crossing *prodh1-4* and *prodh2-2* and was described by Cabassa-Hourton et al. (2016). Surface-sterilized seeds were sown on grids placed on solid half-strength Murashige and Skoog medium (0.8% agar) in Petri dishes and cultivated under a long-day light regime (16 h photoperiod; 90 μmol m^–2^ s^–1^ at 21°C). With the aid of the grids, entire batches of seedlings were transferred to fresh Petri dishes containing sterile water and incubated for 3 days in darkness at 21°C.

For mitochondria isolation, seedlings were sown and grown on soil (16 h photoperiod at 80–100 μmol photons m^–2^ s^–1^ at 21°C).

### Protein Extraction and Mitochondria Enrichment

Seedlings were ground in a cold mortar with 2 mL of grinding buffer [50 mM Tris-HCl pH 7.5, 0.5 mM EDTA, 0.5 mM MgCl_2_, 0.5 mM DTT, 1% (w/v) polyvinylpyrrolidone 30, ± 0.5% (v/v) triton-X-100 and 1 × protease inhibitor cocktail (Sigma P2714)]. Soluble protein extracts were centrifuged for 15 min at 20,000 g at 4°C to remove insoluble plant debris. Supernatants were then desalted into the buffer used for the subsequent enzymatic activity on PD10 Sephadex G25 columns (GE healthcare). Soluble protein concentration was determined according to Bradford (1976) with BSA as standard.

Mitochondria were enriched by several differential centrifugation steps as described by Cabassa-Hourton et al. (2016) from detached leaves of 1-month-old plants that had been incubated in darkness for 5 days prior to mitochondria isolation. Mitochondrial protein concentration was determined according to Lowry et al. (1951) with BSA as standard.

### DNA Constructs and Recombinant Protein Production

Bacterial expression constructs for Arabidopsis ProDH1 and ProDH2 including or excluding the putative mitochondrial transit peptides and with an N-terminal GST tag were generated by amplifying the coding sequence from cDNA with primers that introduce suitable restriction sites (Supplementary Table S1). The resulting PCR products were digested and inserted into pGEX-4T-1 or pGEX-6P-1. Protein expression in *E. coli* BL21(λDE3) star cells was induced with 1 mM IPTG at 18°C overnight. The cells were lysed by sonication in extraction buffer [50 mM Tris pH7.5, 5 mM MgCl_2_, 50 mM KCl, 0.5 mM FAD, 0.5 mM DTT ± 0.1% (w/v) dodecyl maltoside] and the GST fusion proteins were purified with Protino^TM^ Glutathione-Agarose 4B (Macherey-Nagel) according to the recommendations of the supplier. Glutathione was removed from the purified proteins by passage through PD SpinTrap G-25 columns (GE lifescience) equilibrated with extraction buffer.

Expression and purification of 6xHis-tagged Arabidopsis and rice P5CR were performed as described previously (Giberti et al., 2014; Forlani et al., 2015b). Protein concentrations were determined according to Bradford (1976) with BSA as standard. All activity assays were performed with enzymes purified from at least three independent bacterial cultures.

### Enzyme Activity Assays

ProDH activity was calculated from the difference in the rates of DCPIP reduction before and after the addition of 150 mM proline (Huang and Cavalieri, 1979; Schertl et al., 2014). Variable amounts of protein extracts or purified enzymes were incubated at 25°C in 850 μl reaction buffer until a linear decrease of the OD_600_ was observed (typically 2–3 min). Then the reaction was started by adding 150 μl of a 1 M proline solution and the decrease in the OD_600_ was followed until at least 1 min of linear reaction was observed. The final reaction mix contained 100 mM Tris-HCl, pH 7.5, 2.5 mM MgCl_2_, 1 mM KCN, 0.5 mM FAD, 0.5 mM phenazine methosulfate, and 60 μM DCPIP. The activity of P5CR in the physiological forward reaction was calculated from the difference in the rates of NADPH oxidation at pH 7.5 and 25°C in the presence or absence of DL-P5C as described previously (Forlani et al., 2007). DL-P5C was kept as a stock in 1 M HCl and neutralized with 1 M Tris-base immediately before the reaction was started. The final reaction mixture contained 200 mM Tris-HCl, 0.4 mM NADPH, 1 mM DL-P5C and variable amounts of protein extract or purified enzymes. The activity of P5CR in the reverse reaction was calculated from the difference in the rates of NAD^+^ reduction at pH 10 and 25°C before and after the addition of 50 mM proline (from a 1 M stock solution) as described in Forlani and Funck (2020). The final reaction mixture contained 60 mM Na carbonate buffer or 60 mM Glycine-NaOH buffer, pH 10, 10 mM NAD^+^ and variable amounts of protein extract or purified enzymes. In all assays, the amount of enzyme was adjusted to ensure linear reaction rates over at least 1 min. Extinction coefficients of ε_600_ = 19.1 mM^–1^ cm^–1^ for DCPIP (Basford and Huennekens, 1955) and ε_340_ = 6.22 mM^–1^ cm^–1^ for NADH and NADPH were used to calculate specific activities.

## Results

### Expression of Arabidopsis ProDH1 or ProDH2 in *E. coli* Confers the Ability to Catalyze Proline-Dependent DCPIP Reduction, but Not Proline-Dependent NAD^+^ Reduction at High pH

**FIGURE 2:**
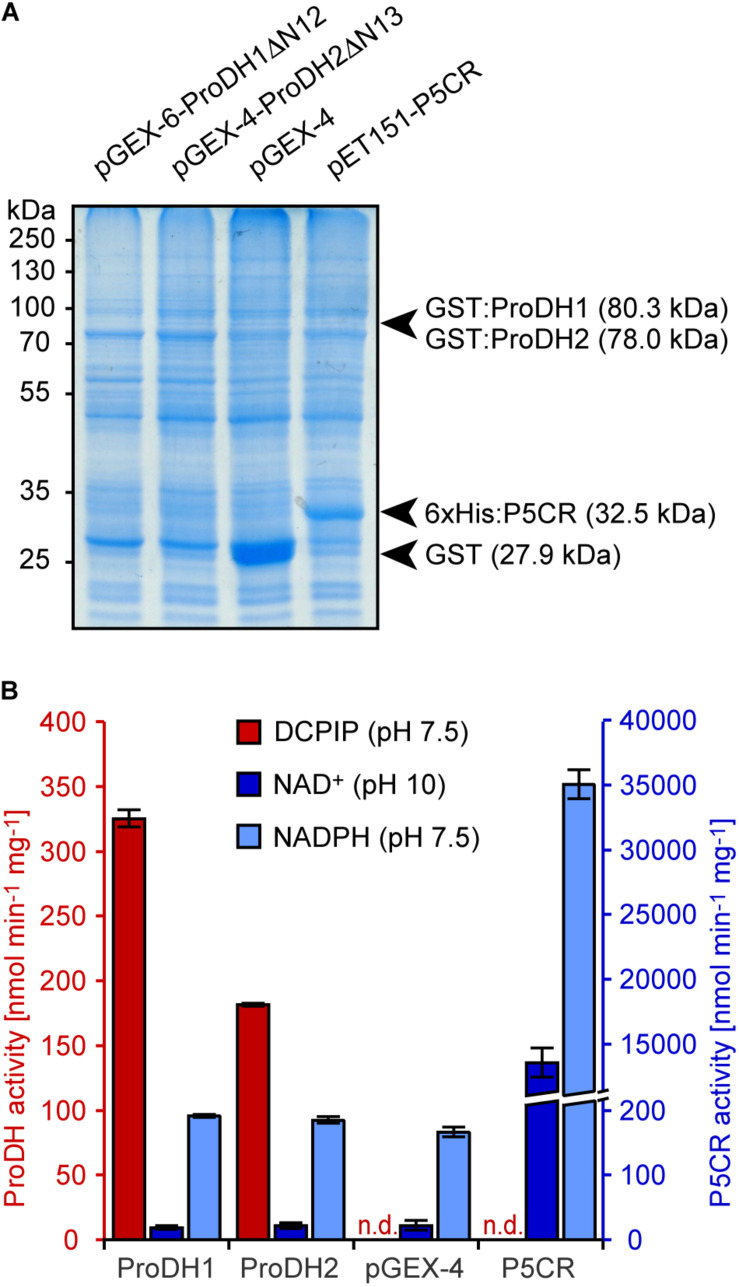
Enzyme activities in bacterial extracts. Soluble protein extracts from *E. coli* cells carrying an empty vector or overexpressing either GST:ProDH1ΔN12, GST:ProDH2ΔN13 or 6xHis:P5CR were used for different enzyme assays. **(A)** Coomassie-stained gel with the positions of molecular weight markers and those expected for the recombinant proteins. **(B)** Proline-dependent 2,6-dichlorophenolindophenol (DCPIP) reduction (red columns) and P5C-dependent NADPH oxidation (light blue columns) were measured at pH 7.5, while proline-dependent NAD^+^ reduction (blue columns) was measured at pH 10. Data are the average (±SD) of technical triplicates. Independent protein preparations gave very similar results; n.d., not detected.

Analysis of mitochondrial protein extracts from Arabidopsis seedlings by mass spectrometry identified N-terminal peptides of ProDH1 and ProDH2 lacking the first 12 and 13 amino acids, respectively, indicating processing of the N-termini during import into mitochondria (Launay et al., 2019; and data not shown). When the deduced mature polypeptides (ProDH1ΔN12 and ProDH2ΔN13) or the pre-proteins of Arabidopsis ProDH1 and ProDH2 were expressed as GST-fusion proteins in *E. coli*, very little soluble protein of the expected size was obtained by expression over night at 10–18°C (Figure 2A). This notwithstanding, crude protein extracts of the *E. coli* cultures expressing ProDH1 or ProDH2, but not from cells carrying an empty vector or expressing Arabidopsis P5CR, showed clearly detectable rates of proline-dependent DCPIP reduction (Figure 2B). These rates were slightly higher in cells expressing the mature forms of ProDH1 and ProDH2 compared to the pre-proteins (data not shown) and therefore, all subsequent experiments were carried out exclusively with the mature proteins. The rates of P5C-dependent NADPH-oxidation at pH 7.5 and of proline-dependent NAD^+^ reduction at pH 10 were very similar in cells carrying the empty vector or expressing ProDH1 or ProDH2, but increased more than 180-fold in cells expressing P5CR (Figure 2B). These observations strongly suggest that only DCPIP reduction can be attributed to ProDH activity and that PutA, the bi-functional ProDH/P5CDH from *E. coli*, was not expressed under our cultivation conditions or was not present in the soluble protein fraction. As expected, P5CR on the other hand seems to mediate both the oxidation of NADPH at pH 7.5 and the reduction of NAD^+^ at pH 10. ProC, the P5CR of *E. coli*, was presumably present in all soluble protein extracts and conferred low rates of proline-dependent NAD^+^ reduction, as well as P5C-dependent NADPH oxidation.

### Purified Recombinant ProDH1 and ProDH2 Do Not Reduce NAD^+^

**FIGURE 3:**
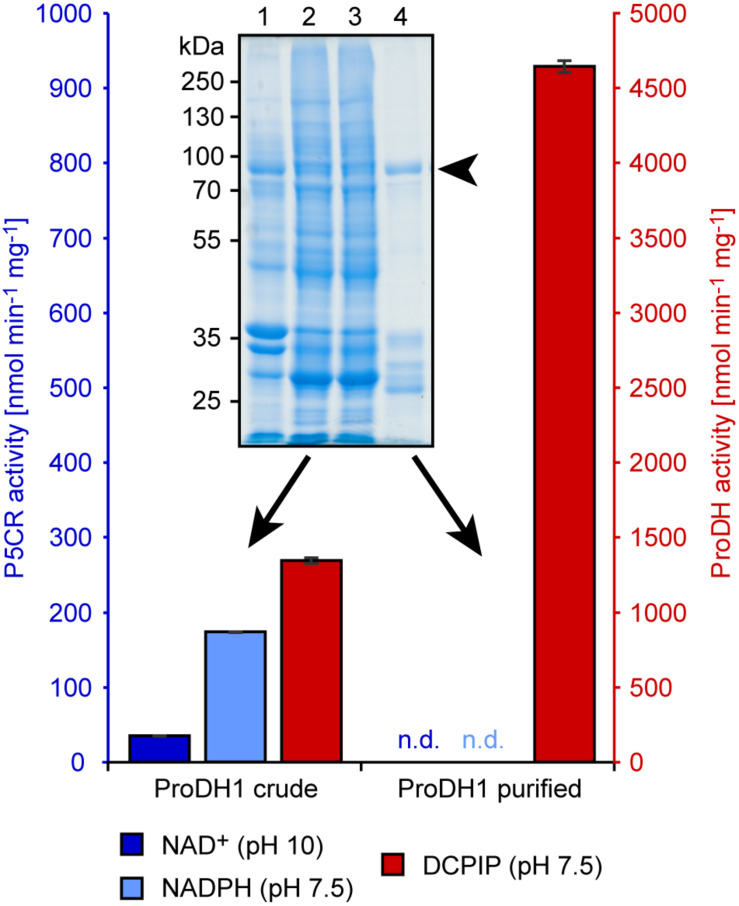
Activities of purified recombinant GST:ProDH1ΔN12. Crude protein extract of bacteria overexpressing GST:ProDH1ΔN12 and affinity-purified GST:ProDH1ΔN12 (indicated by an arrowhead, calculated molecular weight is 80.3 kDa) were assayed for proline-dependent 2,6-dichlorophenolindophenol (DCPIP) reduction and P5C-dependent NADPH oxidation at pH 7.5, as well as for proline-dependent NAD^+^ reduction at pH 10. Note that the scales of the two *y*-axes are in an opposite ratio than in Figure 2. Data are the average (±SD) of technical triplicates. Two further, independent protein preparations gave very similar results; n.d., not detected. The inset shows a Coomassie-stained, denaturing protein gel of the assayed fractions. Lane 1: insoluble proteins, lane 2: soluble extract [in the presence of 0.1% (w/v) dodecyl maltoside], lane 3: flow-through of the glutathione agarose column, lane 4: purified GST:ProDH1ΔN12.

Addition of 0.1% (w/v) dodecyl maltoside to the extraction buffer increased the DCPIP reduction activity in the soluble fraction of ProDH expressing *E. coli* cells and allowed binding of the recombinant proteins to glutathione agarose beads (Figure 3 and Supplementary Figure S1). Proteins with the expected molecular weight of 80.3 kDa for GST:ProDH1ΔN12 and 78.0 kDa for GST:ProDH2ΔN13 were strongly enriched in the glutathione-eluted fraction together with additional smaller proteins. These smaller proteins were recognized by anti-GST antibodies (data not shown), and were different between GST:ProDH1 and GST:ProDH2 preparations, indicating that they are fragments of the recombinant fusion proteins. Overall, a large fraction of the proline-dependent DCPIP reduction activity was lost during the purification, but the specific activity was strongly increased. Neither proline-dependent NAD^+^ reduction at pH 10 nor P5C-dependent NADPH oxidation were consistently detected in the fractions containing the purified ProDHs.

### Purified Recombinant P5CR Reduces NAD^+^ at High pH, but Does Not Reduce DCPIP

**FIGURE 4:**
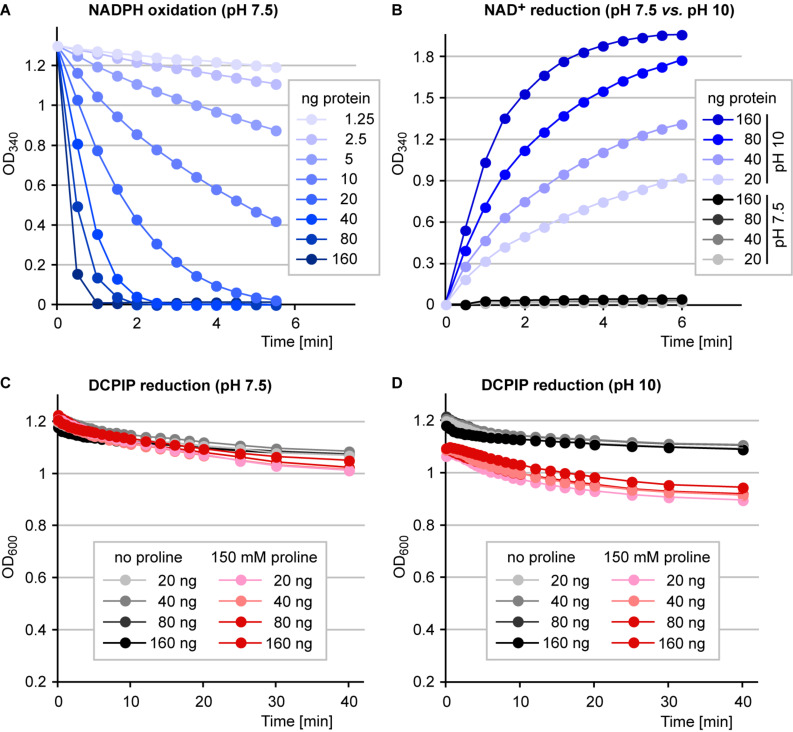
Enzymatic activities of purified P5CR. 6xHis:P5CR was purified and its activity was analyzed photometrically under various conditions. **(A)** P5C-dependent oxidation of NADPH at pH 7.5, the physiological forward-reaction of P5CR, was followed over time with different amounts of enzyme in the assay. **(B)** Proline-dependent reduction of NAD^+^, the reverse reaction of P5CR, was monitored at two different pH values and with different amounts of enzyme. **(C)** Reduction of 2,6-dichlorophenolindophenol (DCPIP) at pH 7.5 with various amounts of enzyme in the presence or absence of proline. **(D)** Reduction of DCPIP at pH 10 with various amounts of enzyme in the presence or absence of proline. All data are from single kinetic analyses. Very similar results were obtained with at least three independently purified enzyme preparations.

We have shown previously, that recombinant Arabidopsis or rice P5CR can easily be purified to electrophoretic homogeneity after overexpression as 6xHis fusion proteins in *E. coli* (Giberti et al., 2014; Forlani et al., 2015b). Purified Arabidopsis P5CR showed rates of P5C-dependent oxidation of NADPH at pH 7.5 and of proline-dependent reduction of NAD^+^ at pH 10 that were proportional to the amount of enzyme in the assay (Figures 4A,B). At physiological pH, however, P5CR did not mediate detectable proline-dependent NAD^+^ reduction. Proline-dependent DCPIP reduction was not detected at either pH 7.5 or pH 10. Only a residual DCPIP reduction that was not proportional to the amount of enzyme was observed in both the presence and absence of proline in the assay (Figures 4C,D). Very similar results were obtained with purified recombinant P5CR from rice (data not shown).

### Soluble Protein Extracts Contain P5CR but Not ProDH Activity

Having demonstrated with purified, recombinant plant proteins that only DCPIP reduction truly reflects ProDH activity, we analyzed the distribution of ProDH and P5CR activities in plant extracts. In soluble protein extracts of plants grown under normal conditions, proline-dependent reduction of DCPIP was not detected, whereas proline-dependent reduction of NAD^+^ at pH 10 and P5C-dependent oxidation of NADPH at pH 7.5 were readily measured in extracts prepared with or without 0.5% (v/v) triton-X-100 (Supplementary Figure S2).

**FIGURE 5:**
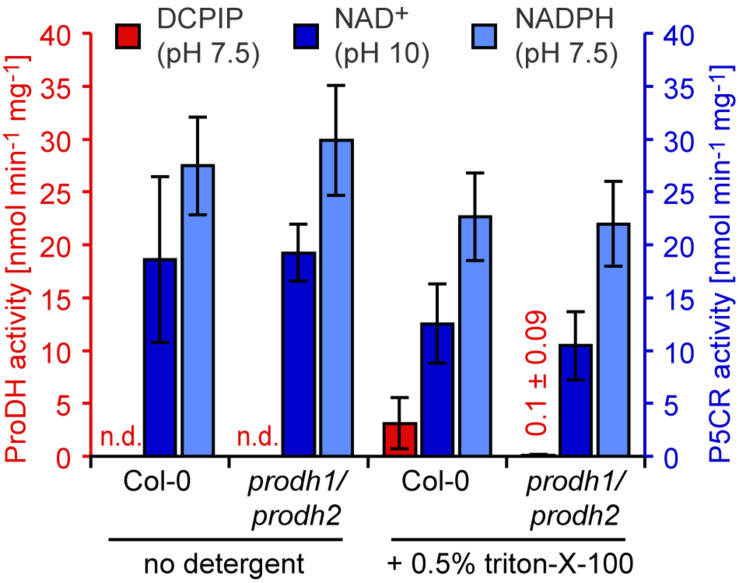
ProDH and P5CR activity levels in Arabidopsis seedlings in response to dark-induced senescence. Twelve-day-old Arabidopsis seedlings grown on 0.5x Murashige and Skoog solid medium were transferred to water and darkness for 3 days to trigger senescence and *ProDH1* and *ProDH2* expression. Enzyme activities were measured in soluble protein extracts prepared with or without 0.5% (v/v) triton-X-100 from wildtype (Col-0) seedlings and *prodh1-4*/*prodh2-2* double mutants. ProDH activity (red bars) was calculated from the proline-dependent reduction of the artificial electron acceptor 2,6-dichlorophenolindophenol (DCPIP) at pH 7.5. P5CR activity (light blue bars) was measured through P5C-dependent oxidation of NADPH at pH 7.5. Reverse P5CR activity (blue bars) was detected based on proline-dependent NAD^+^ reduction at pH 10. Data are means (±SD) of three independent biological replicates. n.d.: ProDH activity not detected.

When Arabidopsis seedlings were placed for 3–5 days in darkness, a condition known to induce ProDH expression (Kovács et al., 2019; Launay et al., 2019), soluble protein extracts without detergent in the extraction buffer did not show proline-dependent DCPIP reduction at pH 7.5 (Figure 5). When the extraction buffer contained 0.5% (v/v) triton-X-100, a low rate of DCPIP reduction (3.1 nmol min^–1^ mg^–1^) was detected in extracts from wildtype seedlings, but this activity was nearly undetectable in extracts from *prodh1-4/prodh2-2* double mutants. In contrast, proline-dependent NAD^+^ reduction at pH 10 as well as P5C-dependent NADPH oxidation at pH 7.5 were detected at similar rates in extracts from wildtype plants and *prodh1-4/prodh2-2* double mutants. The specific activity of proline-dependent NAD^+^ reduction at pH 10 decreased from 18.6 to 12.6 nmol min^–1^ mg^–1^ in extracts of wildtype seedlings when 0.5% (v/v) triton-X-100 was added to the extraction buffer, reflecting a higher total protein concentration in the triton-containing extracts. Very similar results were obtained with 0.1% (w/v) dodecyl maltoside in the extraction buffer and *prodh1-1/prodh2-1* double mutants (data not shown).

### ProDH Activity Is Enriched in Isolated Mitochondria

**FIGURE 6:**
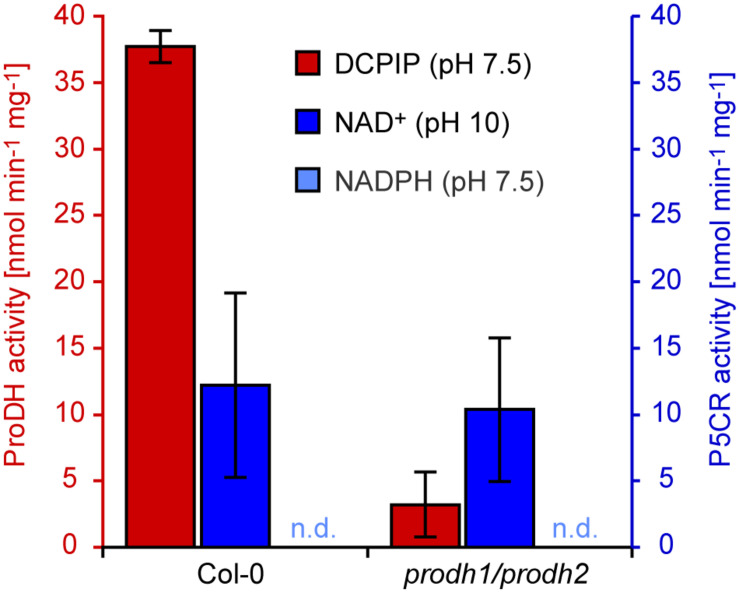
ProDH and P5CR activities in isolated mitochondria. Crude mitochondrial fractions were prepared from Arabidopsis wildtype (Col-0) and *prodh1-4/prodh2-2* double mutant leaves after 5 days of dark treatment. Isolated mitochondria were assayed for proline-dependent 2,6-dichlorophenolindophenol (DCPIP) reduction at pH 7.5 (red columns) and proline-dependent NAD^+^ reduction at pH 10 (blue columns). P5C-dependent NADPH oxidation at pH 7.5 was not detected (n.d.). Data are means (±SD) of three independent preparations of mitochondria.

When mitochondria were partially purified by differential centrifugation of extracts from detached mature leaves kept for 5 days in darkness to induce ProDH expression, proline-dependent DCPIP reduction was detected with specific activity of 37.7 nmol min^–1^ mg^–1^ in wildtype mitochondria, which was more than 10 times higher than in total protein extracts of dark-treated seedlings (Figure 6). When mitochondria were isolated from dark-treated leaves of a *prodh1-4/prodh2-2* double mutant line, the specific activity of proline-dependent DCPIP reduction was less than 10% of the activity in wildtype mitochondria. Proline-dependent NAD^+^ reduction at pH 10 was detected with a rate of 12.2 nmol min^–1^ mg^–1^ and this activity was not significantly different between wildtype mitochondria and mitochondria isolated from leaves of *prodh1-4/prodh2-2* double mutants (10.4 nmol min^–1^ mg^–1^). P5C-dependent NADPH oxidation at pH 7.5 was near or below the detection limit in isolated mitochondria, irrespective of the genotype of the seedlings.

## Discussion

By our detailed comparisons of extracts or purified mitochondria from wildtype plants and *prodh1/prodh2* double mutants, as well as recombinant enzymes expressed in *E. coli*, we demonstrate that both ProDH1 and ProDH2 from Arabidopsis catalyze the proline-dependent reduction of DCPIP in the presence of FAD and phenazine methosulfate, but do not mediate proline-dependent NAD^+^ reduction at pH 10.

When Arabidopsis ProDH1 and ProDH2 were expressed as GST fusion proteins in *E. coli*, expression of the mature proteins without the mitochondrial transit peptides slightly improved expression or solubility of the GST:ProDH fusion proteins, yielding higher specific DCPIP reduction activities in soluble extracts. Additionally, both the forward (P5C-dependent NADPH oxidation) and the reverse (proline-dependent NAD^+^ reduction) activity of P5CR were detected in crude *E. coli* extracts (Figure 2). Because the latter two activities were detected at similar levels in extracts from bacteria harboring an empty vector, they are most likely mediated by ProC, the P5CR from *E. coli*. We did not notice any interference from PutA, the bi-functional ProDH/P5CDH from *E. coli*, in the activity assays. This was to be expected, because the expression of the *putA* gene is under catabolite repression in rich media, and additionally PutA represses its own transcription in the absence of external proline (Nakao et al., 1988). Purification of the GST:ProDH fusion proteins strongly increased the specific activity of proline-dependent DCPIP reduction, whereas the proline-dependent reduction of NAD^+^ at pH 10 was depleted (Figure 3 and Supplementary Figure S1). The specific activities of the purified GST:ProDH1ΔN12 and GST:ProDH2ΔN13 were similar to the activities reported recently for ProDH1ΔN39 and ProDH2ΔN29 expressed as fusion proteins with the maltose binding protein (Fabro et al., 2020).

Conversely, purified P5CRs did not reduce DCPIP, but mediated both proline-dependent NAD^+^ reduction and P5C-dependent NADPH oxidation, depending on the pH of the assay mixture (Figure 4). The same picture was obtained with crude extracts from *E. coli* cells overexpressing Arabidopsis P5CR (Figure 2). Above pH 6.2, P5C dominates in the equilibrium between GSA and P5C (Bearne and Wolfenden, 1995) and in the physiological pH range, the energy of NAD(P)H oxidation drives the reaction catalyzed by P5CR mainly toward proline. At high pH values, the free energy favors the reaction toward P5C because three protons are released during the conversion of proline to P5C. A detailed characterization and discussion of the pH-dependence of the reaction catalyzed by P5CR can be found in Forlani and Funck (2020), where the authors demonstrate that at pH 10.5, the proline-dependent reduction of NAD^+^ by purified P5CR can be used as a specific method for proline quantification.

As reported many times before, both P5C-dependent NADPH oxidation at pH 7.5 and proline-dependent NAD^+^ reduction at pH 10 were readily detectable in crude plant protein extracts, irrespective of the use of mild detergents (Figure 5 and Supplementary Figure S2). These activities were at the same level in *prodh1/prodh2* double mutants as in wildtype plants, demonstrating that they are not related to the native ProDH proteins. Because *P5CR* is an essential gene in Arabidopsis and homozygous mutants are aborted very early during embryo development, it is not possible to use *p5cr* mutants to confirm the origin of these activities (Funck et al., 2012). Detection of proline-dependent DCPIP reduction in crude plant extracts required both the use of a mild detergent in the extraction buffer and the induction of *ProDH1* or *ProDH2* expression (Figure 5 and Supplementary Figure S2). Well-characterized experimental systems for the induction of *ProDH1* and *ProDH2* expression are feeding with external proline or induction of senescence and autophagy by exposure to prolonged periods of darkness (Cabassa-Hourton et al., 2016; Kovács et al., 2019; Launay et al., 2019). We chose dark-induced starvation as the more physiologically relevant treatment. After 3 days of dark induction, the specific activity of proline-dependent DCPIP reduction in extracts of wildtype seedlings was roughly one tenth of the specific P5CR forward or reverse activity. DCPIP reduction was nearly undetectable in extracts from *prodh1/prodh2* double mutants, confirming that this activity is mediated specifically by ProDHs. It has been noted previously that both the *prodh1-4* and the *prodh2-2* mutants contain low levels of native transcripts (Funck et al., 2010) that may explain the residual DCPIP reduction activity even though both ProDH1 and ProDH2 protein levels were below the detection limit of Western blotting (Launay et al., 2019).

Cell fractionation, GFP-tagging and immunodetection in electron micrographs showed that ProDHs are localized in mitochondria (Elthon and Stewart, 1981; Funck et al., 2010; Launay et al., 2019). In mitochondrial preparations from dark-induced wildtype plants, the specific rate of proline-dependent DCPIP reduction was more than three times higher than the proline-dependent reduction of NAD^+^, whereas the latter activity was typically at least fourfold higher than DCPIP reduction in total protein extracts (Figures 5, 6). Again, specifically the DCPIP reduction activity was lower in mitochondria isolated from *prodh1/prodh2* double mutants, confirming that ProDHs mediate this activity. In contrast, NAD^+^ reduction was not affected and can therefore not be mediated by ProDHs. It was unexpected to detect proline-dependent NAD^+^ reduction at pH 10 in isolated mitochondria, because analysis of plants expressing a P5CR:GFP fusion protein did not provide any evidence for mitochondrial import or association of P5CR in Arabidopsis (Funck et al., 2012). However, co-fractionation of P5CR activity with organelles, namely plastids, has been observed previously in other plant species (Rayapati et al., 1989; Szoke et al., 1992; Murahama et al., 2001). The exclusive detection of the reverse activity of P5CR in isolated mitochondria could be explained by a high content of P5CDH, which is also a mitochondrial enzyme in Arabidopsis (Deuschle et al., 2001). An excess of P5CDH will compete with P5CR for P5C and since P5CDH accepts both NAD^+^ or NADP^+^ as electron acceptor, any NADP^+^ formed by P5CR would be instantly re-reduced by P5CDH (Forlani et al., 1997).

We are aware that numerous previous publications have assigned proline-dependent NAD^+^ reduction at pH 9 or higher in plant extracts or recombinant proteins to ProDH. Our data clearly demonstrate that the conclusions based on this erroneous assignment may be at least partially wrong or misleading. It is beyond the scope of this article to try to provide a comprehensive list of these publications or to provide a detailed discussion of previous conclusions that may no longer be valid. However, we urge all researchers in the field of proline metabolism to be aware of this pitfall and help to ensure, both in their roles as researchers and as reviewers, that only suitable enzyme assays will be applied in future studies.

## Data Availability Statement

The raw data supporting the conclusions of this article will be made available by the authors, without undue reservation, to any qualified researcher.

## Author Contributions

DF initiated the study, performed assays with recombinant ProDH and plant protein extracts, and drafted the manuscript. GF performed the assays with recombinant P5CR. SL, CC-H, and AS performed the experiments with plant extracts and isolated mitochondria. All authors contributed to the data evaluation and the final manuscript.

## Conflict of Interest

The authors declare that the research was conducted in the absence of any commercial or financial relationships that could be construed as a potential conflict of interest.

## Supplementary Material

Supplementary Figure 1 Specific activity of purified recombinant GST:ProDH2ΔN13. Crude protein extract of bacteria overexpressing GST:ProDH2ΔN13 and affinity-purified GST:ProDH2ΔN13 (indicated by an arrowhead, calculated molecular weight is 78.0 kDa) were assayed for proline-dependent 2,6-dichlorophenolindophenol (DCPIP) reduction (red columns) and P5C-dependent NADPH oxidation at pH 7.5 (light blue columns), as well as for proline-dependent NAD^+^ reduction at pH 10 (dark blue columns). Note that the scales of the two *y*-axes are in an opposite ratio than in Figure 2. Data are the average (±SD) of technical triplicates. Two further, independent protein preparations gave very similar results; n.d., not detected. The inset shows a Coomassie-stained, denaturing protein gel of the assayed fractions. Lane 1: soluble extract [in the presence of 0.1% (w/v) dodecyl maltoside], lane 2: flow-through of the glutathione agarose column, lane 3: purified GST:ProDH2ΔN13.

Supplementary Figure 2 ProDH and P5CR activities in Arabidopsis seedlings. Twelve-days-old Arabidopsis wildtype (Col-0) seedlings grown on 0.5x Murashige and Skoog solid medium were used to prepare soluble protein extracts either with or without 0.5% triton-X-100. ProDH activity, assayed as proline-dependent reduction of 2,6-dichlorophenolindophenol (DCPIP) at pH 7.5, was not detected (n.d.). P5CR activity (light blue bars) was measured as P5C-dependent oxidation of NADPH at pH 7.5. Reverse P5CR activity (blue bars) was detected as proline-dependent NAD^+^ reduction at pH 10. Data are means (±SD) of three independent batches of seedlings.

Supplementary Table 1 Primers used for cloning of ProDH expression constructs.
