# Supplementary material for: Appropriate Activity Assays Are Crucial for the Specific Determination of Proline Dehydrogenase and Pyrroline-5-Carboxylate Reductase Activities
Source: Front Plant Sci. 2020 Dec 23;11:602939. doi: 10.3389/fpls.2020.602939 (PMC7785524; doi:10.3389/fpls.2020.602939)
Supplement: Supplementary Figure 1 — Specific activity of purified recombinant GST:ProDH2ΔN13. Crude protein extract of bacteria overexpressing GST:ProDH2ΔN13 and affinity-purified GST:ProDH2ΔN13 (indicated by an arrowhead, calculated molecular weight is 78.0 kDa) were assayed for proline-dependent 2,6-dichlorophenolindophenol (DCPIP) reduction (red columns) and P5C-dependent NADPH oxidation at pH 7.5 (light blue columns), as well as for proline-dependent NAD+ reduction at pH 10 (dark blue columns). Note that the scales of the two y-axes are in an opposite ratio than in Figure 2. Data are the average (±SD) of technical triplicates. Two further, independent protein preparations gave very similar results; n.d., not detected. The inset shows a Coomassie-stained, denaturing protein gel of the assayed fractions. Lane 1: soluble extract [in the presence of 0.1% (w/v) dodecyl maltoside], lane 2: flow-through of the glutathione agarose column, lane 3: purified GST:ProDH2ΔN13. [file Data_Sheet_1.PDF]

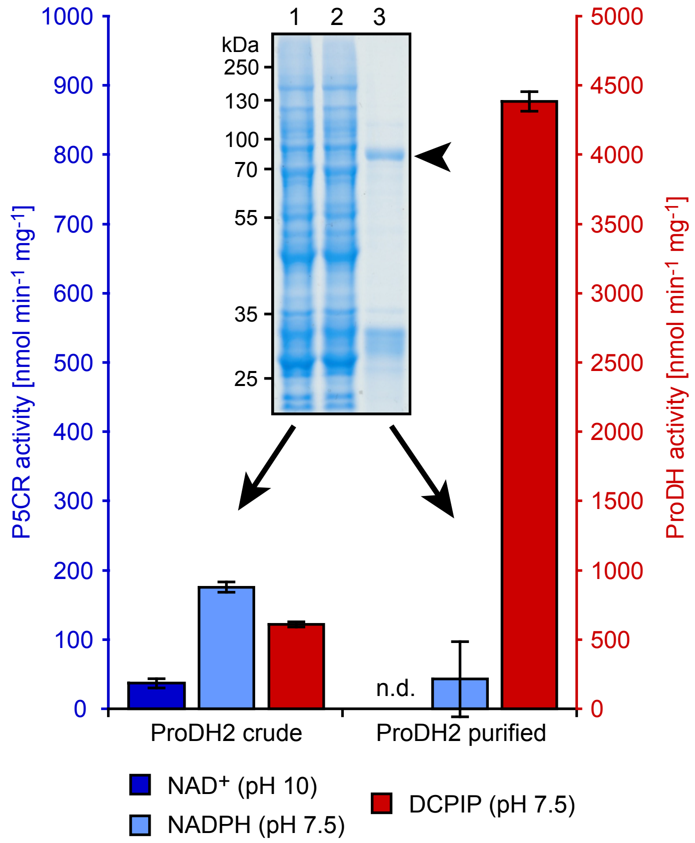

Supplementary Figure S1: Specific activity of purified recombinant GST:ProDH2ΔN13
Crude protein extract of bacteria overexpressing GST:ProDH2ΔN13 and affinity-purified GST:ProDH2ΔN13 (indicated by an arrowhead, calculated molecular weight is 78.0 kDa) were assayed for proline-dependent 2,6-dichlorophenolindophenol (DCPIP) reduction (red columns) and P5C-dependent NADPH oxidation at pH 7.5 (light blue columns), as well as for proline-dependent NAD^+ reduction at pH 10 (dark blue columns). Note that the scales of the two y-axes are in an opposite ratio than in Fig. 2. Data are the average of technical triplicates (± SD). Two further, independent protein preparations gave very similar results; n.d., not detected. The inset shows a Coomassie-stained, denaturing protein gel of the assayed fractions. Lane 1: soluble extract [in the presence of 0.1% (w/v) dodecyl maltoside], lane 2: flow-through of the glutathione agarose column, lane 3: purified GST:ProDH2ΔN13.
