# Supplementary material for: Appropriate Activity Assays Are Crucial for the Specific Determination of Proline Dehydrogenase and Pyrroline-5-Carboxylate Reductase Activities
Source: Front Plant Sci. 2020 Dec 23;11:602939. doi: 10.3389/fpls.2020.602939 (PMC7785524; doi:10.3389/fpls.2020.602939)
Supplement: Supplementary Figure 2 — ProDH and P5CR activities in Arabidopsis seedlings. Twelve-days-old Arabidopsis wildtype (Col-0) seedlings grown on 0.5x Murashige and Skoog solid medium were used to prepare soluble protein extracts either with or without 0.5% triton-X-100. ProDH activity, assayed as proline-dependent reduction of 2,6-dichlorophenolindophenol (DCPIP) at pH 7.5, was not detected (n.d.). P5CR activity (light blue bars) was measured as P5C-dependent oxidation of NADPH at pH 7.5. Reverse P5CR activity (blue bars) was detected as proline-dependent NAD+ reduction at pH 10. Data are means (±SD) of three independent batches of seedlings. [file Data_Sheet_2.PDF]

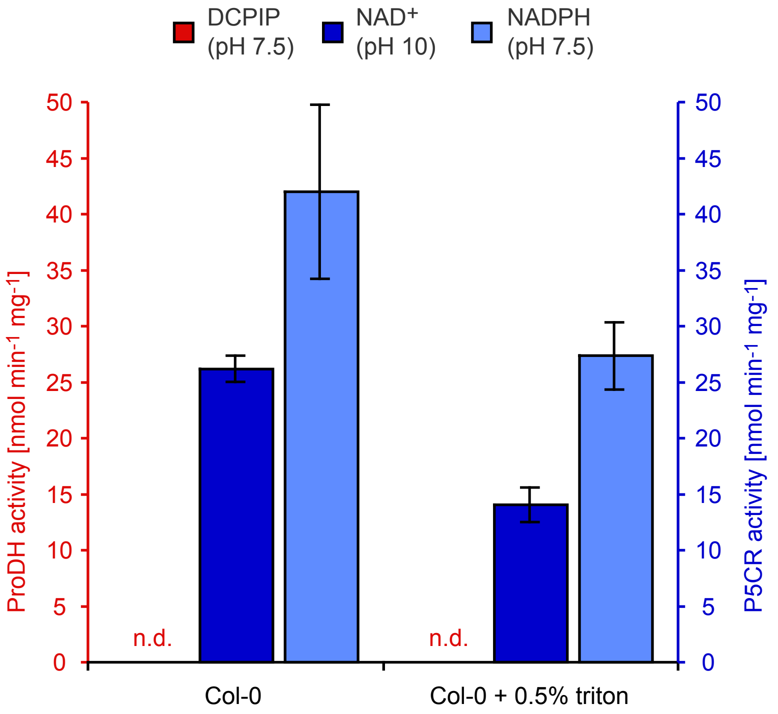

Supplementary Figure 2: ProDH and P5CR activities in Arabidopsis seedlings

Twelve-day-old Arabidopsis wildtype (Col-0) seedlings grown on 0.5x Murashige and Skoog solid medium were used to prepare soluble protein extracts either with or without 0.5% (v/v) triton-X-100. ProDH activity, assayed as proline-dependent reduction of 2,6-dichlorophenolindophenol (DCPIP) at pH 7.5, was not detected (n.d.). P5CR activity (light blue bars) was measured as P5C-dependent oxidation of NADPH at pH 7.5. Reverse P5CR activity (blue bars) was detected as proline-dependent NAD^+ reduction at pH 10. Data are means (± SD) of three independent batches of seedlings.
