# Supplementary material for: Appropriate Activity Assays Are Crucial for the Specific Determination of Proline Dehydrogenase and Pyrroline-5-Carboxylate Reductase Activities
Source: Front Plant Sci. 2020 Dec 23;11:602939. doi: 10.3389/fpls.2020.602939 (PMC7785524; doi:10.3389/fpls.2020.602939)
Supplement: Supplementary Table 1 — Primers used for cloning of ProDH expression constructs. [file Data_Sheet_3.PDF]

Supplementary Table S1: Primers used for cloning of ProDH expression constructs

Restriction sites used for inserting the PCR products into the expression vectors are shown in lowercase letters, the first codons belonging to *ProDH1* or *ProDH2* and stop codons are indicated by bold letters.

**Primer name**	**Sequence (5'→ 3')**	**Expression vector**
ProDH1(fl)-F	Ggaattc**ATG**GCAACCCGTCTTCTC	pGEX-4T-1
ProDH1(XhoI)-R	Gctcgag**TTA**CGCAATCCCGGCGATTAATCTC	
ProDH1(ΔN12)-F	GCggatcc**CGA**TCTTACCGTTTACC	pGEX-6P-1
ProDH1(EcoRI)-R	GCgaattc**TTA**CGCAATCCCGGC	
ProDH2(fl)-F	Ggaattc**ATG**GCAAACCGTTTCCTC	pGEX-4T-1
ProDH2(ΔN13)-F	Ggaa**ttc**TCCACCGTGAGTCCCGTC	
ProDH2-R	Gctcgag**TCA**CCAAGCCATAACTC	
